# CD10 and Das1: a biomarker study using immunohistochemistry to subtype gastric intestinal metaplasia

**DOI:** 10.1186/s12876-022-02268-z

**Published:** 2022-04-21

**Authors:** Athanasios Koulis, Natasha Di Costanzo, Catherine Mitchell, Stephen Lade, David Goode, Rita A. Busuttil, Alex Boussioutas

**Affiliations:** 1 Upper Gastrointestinal Translational Research Laboratory, Peter MacCallum Cancer Centre, Melbourne, Australia; 2 The Sir Peter MacCallum Department of Oncology, The University of Melbourne, Melbourne, Australia; 3 Department of Pathology, Peter MacCallum Cancer Centre, Melbourne, Australia; 4 Computational Cancer Biology Program, Peter MacCallum Cancer Centre, Melbourne, Australia; 5 Department of Medicine, Royal Melbourne Hospital, Melbourne, Australia; 6 Upper Gastrointestinal Translational Research Laboratory, Peter MacCallum Cancer Centre, 305 Grattan Street, Parkville, VIC 3050 Australia

**Keywords:** Intestinal metaplasia subtypes, Risk of progression, Gastric cancer, Gene expression profiling, Biomarkers, CD10, Das1, Immunohistochemistry, Logistic regression model, Digital quantification

## Abstract

**Background:**

Intestinal metaplasia (IM) is considered a key pivot point in the Correa model of gastric cancer (GC). It is histologically subtyped into the complete and incomplete subtypes, the latter being associated with a greater risk of progression. However, the clinical utility of IM subtyping remains unclear, partially due to the absence of reliable defining biomarkers.

**Methods:**

Based on gene expression data and existing literature, we selected CD10 and Das1 as candidate biomarkers to distinguish complete and incomplete IM glands in tissues from patients without GC (IM-GC) and patients with GC (IM + GC). Immunohistochemical staining of individually subtyped IM glands was scored after blinding by two researchers using tissue belonging to both IM-GC and IM + GC patients. Whole tissue Das1 staining was further assessed using digital image quantification (cellSens Dimension, Olympus).

**Results:**

Across both cohorts CD10 stained the IM brush border and was shown to have a high sensitivity (87.5% and 94.9% in IM-GC and IM + GC patients respectively) and specificity (100.0% and 96.7% respectively) with an overall AUROC of 0.944 for complete IM glands. By contrast Das1 stained mainly goblet cells and the apical membrane of epithelial cells, mostly of incomplete IM glands with a low sensitivity (28.6% and 29.3% in IM-GC and IM + GC patients respectively) but high specificity (98.3% and 85.1% respectively) and an overall AUROC of 0.603 for incomplete IM glands. A combined logistic regression model showed a significant increase in AUROC for detecting complete IM glands (0.955 vs 0.970). Whole tissue digital quantification of Das1 staining showed a significant association with incomplete IM compared to complete IM, both in IM-GC and in IM + GC patients (*p* = 0.016 and *p* = 0.009 respectively, Mann–Whitney test and unpaired t test used). Additionally, complete IM in IM + GC patients exhibited significantly more Das1 staining than in IM-GC patients (*p* = 0.019, Mann–Whitney test).

**Conclusions:**

These findings suggest that CD10 is an outstanding biomarker for complete IM and Das1 may be useful as a secondary biomarker for IM glands at greater risk of progression irrespective of IM subtype. Overall, the clinical use of these biomarkers could lead to improved patient stratification and targeted surveillance.

**Supplementary Information:**

The online version contains supplementary material available at 10.1186/s12876-022-02268-z.

## Introduction

Gastric cancer (GC) is the fifth most common and third most lethal cancer globally [1]. Patients with GC are often asymptomatic, with presentation occurring at advanced stage [2], and a low 5-year survival rate in most countries (18–32%, [3, 4]). Countries with population screening programs such as Japan and South Korea have significantly higher survival rates (> 62% and > 75% respectively) [5, 6], with treatment of early GC associated with a 5-year survival rate of over 90% [7, 8].

The Correa model describes histologically defined conditions initiated by *H. pylori* infection, from chronic gastritis (ChG) to atrophic gastritis, intestinal metaplasia (IM), dysplasia and finally to the intestinal type of GC [9]. Successful *H. pylori* eradication treatment in the early stages of this cascade can reverse the process [10, 11] but in a subset of IM patients, eradication does not prevent them from progressing to GC suggesting that IM is a key point in gastric carcinogenesis [11].

IM is found in approximately 25% of the global population [12] and in certain populations 1 in 39 individuals with IM is predicted to progress to GC within 20 years [13]. IM is frequently classified histologically into two major subtypes: (i) complete IM which resembles the small intestine with goblet cells, Paneth cells, enterocytes and a brush border and (ii) incomplete IM which more closely approximates colonic epithelium with goblet cells, enterocytes and irregular sized mucin droplets [14].

Case control studies and meta-analyses have shown incomplete IM to be associated with a greater risk of progression to GC in comparison to complete IM [15–17] suggesting value in accurately assigning a subtype of IM. However, guidelines for including incomplete IM as a factor for patient follow up differ between countries. In the UK, IM subtype is not included as a risk factor in the follow-up guidelines by the British Society of Gastroenterology (BSG) [18]. By contrast, the European guidelines for the management of epithelial precancerous conditions and lesions in the stomach (MAPS II) recommend endoscopic surveillance within 3 years if incomplete IM is present in a single location as part of a multifactorial consideration that includes family history of GC and persistent *H. pylori* infection [19].

Patient stratification and targeted surveillance of IM patients at risk of progression would benefit from highly sensitive and specific biomarkers for IM subtypes. This current study explored the potential of two biomarkers for IM, CD10 and Das1. CD10 is a brush border protein with 100% specificity for normal small intestinal mucosa and is absent in the colon [20]. Das1 is a monoclonal antibody which binds colon epithelial protein (CEP). It also reacts with Barrett’s epithelium as well as gastric IM [21, 22] but not with normal stomach nor small intestinal tissue [23]. Previously an association between Das1 and incomplete IM was shown as well as higher reactivity to IM in IM patients with concurrent GC than in IM patients with no GC (*p* < 0.0001, [22]).

In this study, we investigated the utility of CD10 and Das1 to help objective assessment of IM subtype. Additionally, we aimed to find a marker to indicate higher risk of progression in complete IM.

## Materials and methods

### Samples and patient details

The Molecular Analysis for Upper Gastrointestinal Cancer (MAUGIC) cohort consists of gastric and oesophageal cancer patients collected from 1999 to 2020. At the time of gastric resection, tumour and non-malignant tissue samples (at least 2 cm away from tumour) were collected. FFPE blocks containing non-malignant tissue with evidence of IM as characterised by the in-house pathologist were identified for use in this study. IM samples from a second cohort of IM patients, part of an ongoing screening and surveillance program, but with no evidence of GC were collected for this study and defined as the IM-GC cohort.

Written informed consent was obtained and ethical approval was acquired from the Institutional Review Boards of the individual hospitals that participated in the study (HREC ref 2005.075 and 12/25). Clinical details of patients involved in this study are described in Additional file 1: Table S1.

### Subtyping of intestinal metaplasia

H&E stained gastric IM tissue samples collected post-endoscopy/gastrectomy were subtyped at the time of collection by the in-house pathologist. They were subtyped again prior to the current study independently by two pathologists (CM and SL) with further discussion if a consensus decision was required. IM tissue was classified as either complete or incomplete (this included both incomplete and mixed) types (Additional file 1: Methods, Table S2).

Individual IM glands were subtyped as complete or incomplete with principal criteria being the presence of a brush border and gland morphology. As a result, subtyping of glands was based on the upper part of the gland as lower regions often lack a brush border (Additional file 1: Figure S1).

### Gene expression profiling

RNA was extracted (RNeasy kit; Qiagen) from fresh frozen macro-dissected tissue of IM-GC patients (n = 14, Additional file 1: Table S3) and profiled using Affymetrix U133 plus 2 arrays as per manufacturer’s instructions [24] (GEO accession: GSE160116).

Unsupervised hierarchical clustering of IM-GC samples based on expression from Affymetrix microarray data of key genes associated with complete IM/small intestine or incomplete IM/colon was carried out using the pheatmap package in R. Single sample gene set enrichment analysis (ssGSEA) was carried out using the GSVA package in R [25]. The KEGG pathway database was downloaded from the Molecular Signatures Database (www.gsea-msigdb.org/gsea/msigdb/index.jsp).

### Immunohistochemistry

Immunohistochemistry (IHC) was performed on sequential 4 μm FFPE sections. Anti-CD10 (clone 56C6, Abcam, cat# ab951) staining was carried out using both single IHC and as part of a multiplex IHC panel (Additional file 1: Methods, [26]). Multiplexed IHC stained sections were scanned and visualised on a VECTRA® imaging system with inForm® software (PerkinElmer). Pseudo-pathology images of CD10 staining were exported as TIFF files and single IM glands were scored blindly as CD10 positive or negative by two experienced researchers (AB and RB, Additional file 1: Figure S2).

Das1 staining was performed with overnight incubation of the primary antibody (7E12H12, Merck, cat# MABC530; dilution 1:200) at 4 °C followed by incubation with the EnVision + System/anti-mouse HRP reagent and visualised with DAB chromogen. Slides were scanned on a VS120 slide scanner microscope, imaged using cellSens Dimension software (Olympus) and matched IM glands from previously annotated H&E images were scored blindly as positive or negative (AB and RB).

### Digital quantification of whole tissue Das1 staining

Regions rich in IM tissue or adjacent ChG as a control were investigated for Das1 staining. Digital quantification of Das1 staining was carried out using cellSens Dimension to determine the fraction area positive for Das1 staining (Additional file 1: Figure S3).

### Statistical analysis

For gene and pathway analyses of microarray data, multiple test correction was performed using the Benjamini–Hochberg method and significance was set at *p* < 0.05. Differential gene expression was carried out with the limma package in R, with differential expression set at log2 fold change > 0.6 or < − 0.6 and adjusted FDR at *p* < 0.05.

Area under the receiver operating characteristic (AUROC) was calculated using the ROCR package in R. Analysis of Das1 staining was performed using an unpaired t-test (Gaussian distribution of data) or a Mann–Whitney test (non-Gaussian distribution of data) where appropriate. Graphs showing ROC curve and fraction of Das1 positive staining were created using R (pROC package) and GraphPad Prism respectively. A *p*-value of < 0.05 was considered significant.

## Results

### Histo-molecular profiling of IM

We hypothesised that histologically defined gastric complete IM would be rich in expression of genes whose protein products have previously been shown to be associated with complete IM or the small intestine and that histologically defined incomplete IM would be high in expression of genes whose protein products have previously been shown to be associated with incomplete IM or the colon. Our objective was first to identify genes that could be used to molecularly subtype IM samples from Affymetrix microarray data and second to validate potential gene product biomarkers on histologically defined complete and incomplete single IM glands. We initially chose single gland and not whole tissue validation as this would correspond to the highest possible resolution of histological subtyping.

Previous studies have shown exclusive expression of brush border markers such as CD10 (*MME* gene) and IAP (*ALPI* gene) [27–29] in the complete subtype of IM as well as higher expression of CDX2 when compared to the incomplete subtype [30]. By contrast higher expression of CD24 has been described in Type II incomplete IM [31]. To carry out an exploratory molecular-based subgrouping of macro-dissected IM-GC samples with the available Affymetrix microarray data, two additional genes, MUC12 and CDX1, were chosen that have previously been shown to be tissue enriched in the colon when compared to the small intestine [32, 33].

**Fig. 1:**
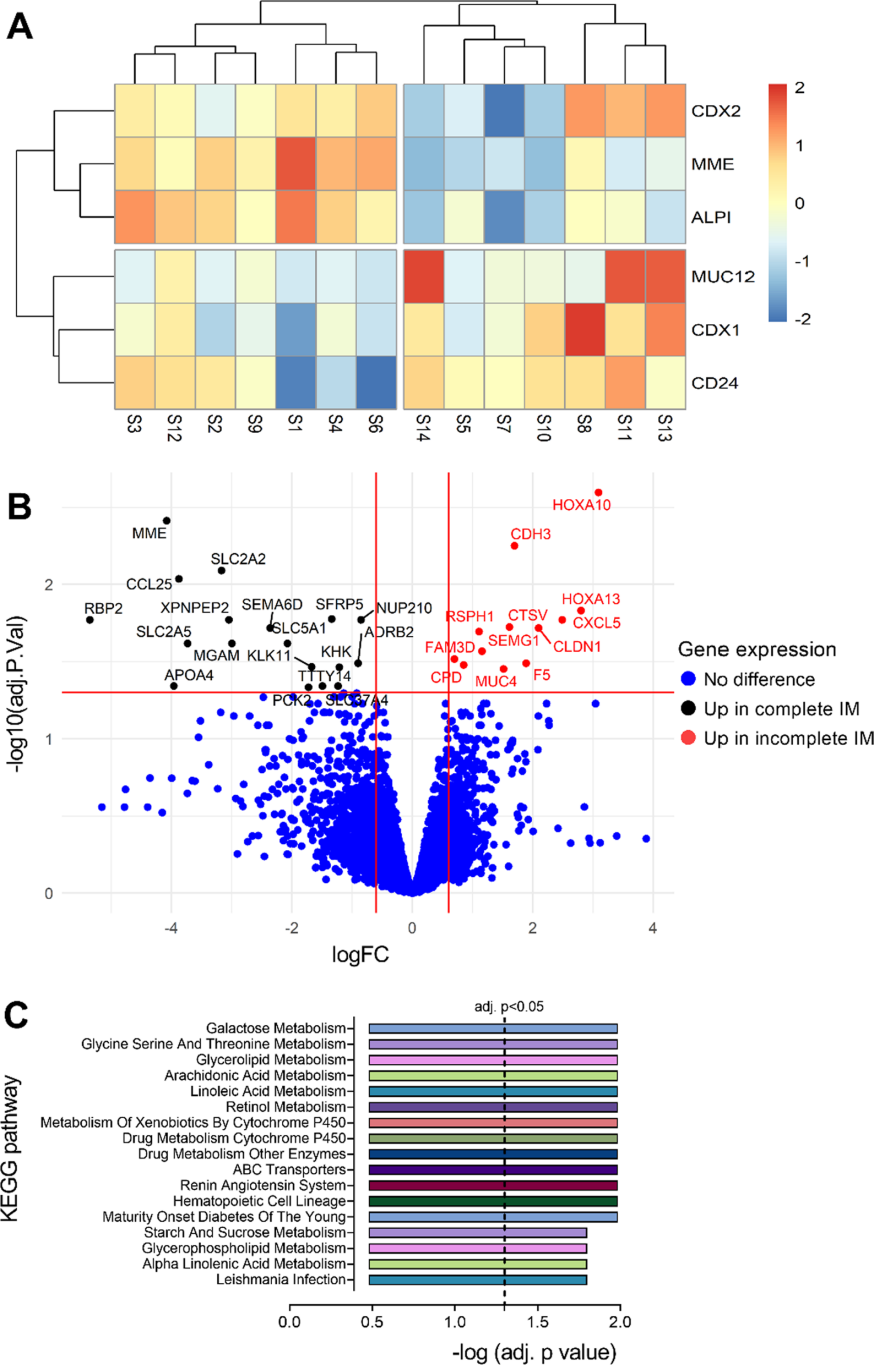
Gene expression analysis of IM samples in patients without cancer (IM-GC). **A** Heatmap showing unsupervised clustering of samples using *ALPI*, *CD24*, *CDX1*, *CDX2*, *MME*, and *MUC12* to subgroup samples (n = 14). Expression levels have been standardised (centered and scaled) within rows for visualization. Legend shows z score**.** Cluster 1 represents complete IM and cluster 2 represents incomplete IM samples. Samples S8 and S12 were removed from these 2 clusters as they likely represent mixed IM. **B** Volcano plot showing differentially expressed genes (logFC > 0.6 or < − 0.6 with FDR adjusted *p* < 0.05) between complete and incomplete IM. Probes with no gene names and differentially expressed probes/genes with duplicates removed. **C** Bar plot showing KEGG pathways [34] enriched in molecularly subtyped complete IM using single sample gene set enrichment analysis (ssGSEA). To calculate statistical significance, the Wilcoxon rank sum test followed by multiple test correction (Benjamini–Hochberg method) was used. No enriched pathways were detected in incomplete IM. Differential gene expression and ssGSEA were performed using the limma and GSVA packages in R

Using the above 6 gene signature, unsupervised hierarchical clustering of IM-GC samples produced two main clusters (Fig. 1A): cluster C1 containing samples with high expression of *CDX2*, *MME* and *ALPI* and cluster C2 containing samples with relatively higher expression of *MUC12*, *CD24* and *CDX1*. Samples S12 (from C1) and S8 (from C2) were classified as molecularly subtyped mixed IM due to the relatively high expression of all 6 target genes and the remaining samples were defined either as molecularly subtyped complete IM (C1 without sample S12) or incomplete IM (C2 without sample S8).

### Differential gene expression and pathway analysis of IM-GC samples

To gain insight into how complete and incomplete IM might differ overall at the gene expression level and use this information to identify an optimal subtype biomarker, differential gene expression analysis was carried out (Fig. 1B, Additional file 1: Table S4). A total of 18 and 12 genes were over-expressed (log2 fold change > 0.6 or < − 0.6 with adjusted FDR at *p* < 0.05) in complete and incomplete IM, respectively, and comprised the differentially expressed gene (DEG) signature. Molecular based IM subtyping was further confirmed by performing unsupervised hierarchical clustering of all IM-GC samples, including the two mixed IM samples, with the DEG signature (Additional file 1: Figure S4). Overall, the complete IM gene list was enriched in small intestine specific genes (*RBP2*, *MME*, *XPNPEP2*) and others related to carbohydrate digestion (*APOA4*, *SLC2A5*, *SLC2A2*, *MGAM* and *KHK*) confirming the strong small intestinal-like characteristics of these samples. There was also a highly enriched chemokine, *CCL25*. The incomplete IM gene list contained genes normally expressed in the colon (*HOXA10* and *HOXA13*) and a chemokine, *CXCL5*. Additionally, two other GC associated genes were also present in the incomplete IM list (*CLDN1* and *CDH3*).

To further determine whether the complete IM samples were relatively enriched in small intestine associated pathways compared to the incomplete IM samples, ssGSEA using the KEGG pathway database was performed. Eighteen pathways were significantly enriched (adjusted *p* < 0.05, Wilcoxon rank sum test with Benjamini–Hochberg correction) in complete IM but none in incomplete IM (Fig. 1C, Additional file 1: Table S5). Enriched pathways were mainly associated with carbohydrate and lipid metabolism suggesting that complete IM was indeed enriched in small intestine associated processes.

### CD10 as a biomarker for single complete IM glands

**Fig. 2:**
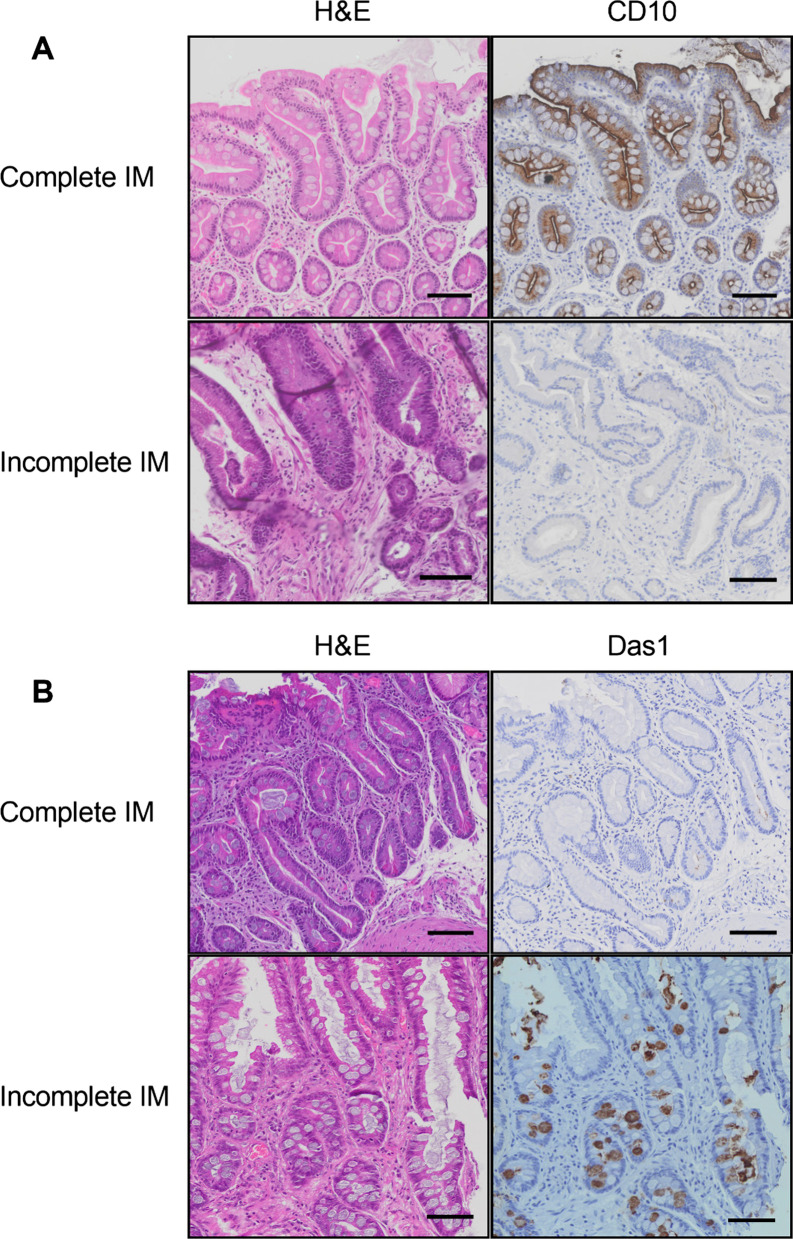
Representative Anti-CD10 and Das1 staining on complete and incomplete intestinal metaplasia tissue. **A** Complete IM tissue stained positive for CD10 but incomplete IM tissue was negative for CD10. **B** Complete IM tissue was negative for Das1 whereas incomplete IM tissue was positive for Das1 staining. Scale bar: 100 μm

Given its highly significant difference in gene expression levels between complete and incomplete IM samples, the gene product of the *MME* gene, CD10, was chosen as a candidate biomarker for complete IM. Initial validation of the anti-CD10 antibody was accomplished with IHC staining of complete and incomplete IM samples (Fig. 2A).

Next, IM samples representing both the IM-GC and IM + GC cohorts were stained with CD10 using immunofluorescence staining. Single gland analysis demonstrated that CD10 is highly sensitive (91.1%) and specific (97.8%) for detecting complete IM glands (Table 1) with high PPV (98.2%) and NPV (89.1%). Further stratification of the samples based on cohorts representing varying risk of progression to GC (where IM-GC is lowest risk and IM + GC is highest risk), showed CD10 had an increasing sensitivity for detecting complete IM glands (from 87.5% in IM-GC patients to 94.9% in IM + GC patients). The reverse trend was observed for specificity with 100.0% in IM-GC patients and 96.7% in IM + GC patients. PPV was above 96% in both cohorts and NPV increased from 80.0% in the IM-GC cohort to 95.1% in the IM + GC cohort.

**Table 1:** Sensitivity and specificity of CD10 and Das1 for individual complete and incomplete intestinal metaplastic glands

Biomarker	IM gland subtype	N^0^ of glands	N^0^ + ve glands	N^0^ − ve glands	Sensitivity	Specificity	PPV/	AUROC
					(95% CI)^a^	(95% CI)^a^	NPV	
*CD10*								
All cohorts								
	Complete	123	112	11	91.1%	97.8%	98.2%/	0.944
	Incomplete	92	2	90	(84.6–95.5%)	(92.4–99.7%)	89.1%	
IM-GC								
	Complete	64	56	8	87.5%	100.0%	100.0%/	0.938
					(76.9–94.5%)	(89.1–100.0%)	80.0%	
	Incomplete	32	0	32				
IM + GC								
	Complete	59	56	3	94.9%	96.7%	96.6%/	0.958
	Incomplete	60	2	58	(85.9–98.9%)	(88.5–99.6%)	95.1%	
*Das1*								
All cohorts								
	Complete	127	11	116	29.2%	91.3%	71.8%/	0.603
					(20.3–39.3%)	(85.0–95.6%)	63.0%	
	Incomplete	96	28	68				
IM-GC								
	Complete	60	1	59	28.6%	98.3%	85.7%/	0.635
	Incomplete	21	6	15	(11.3–52.2%)	(91.1–100.0%)	79.7%	
IM + GC								
	Complete	67	10	57	29.3%	85.1%	68.8%/	0.572
	Incomplete	75	22	53	(19.4–40.1%)	(74.3–92.6%)	51.8%	

^a^Confidence intervals for sensitivity and specificity are Clopper–Pearson confidence intervals; *PPV*, Positive Predictive Value; *NPV*, Negative Predictive Value; *AUROC*, Area Under Receiver Operating Characteristic (ROCR package in R)

### Das1 as a biomarker for single incomplete IM glands

Given complete IM has less propensity to progress to cancer than incomplete IM, it is important to try and identify markers that may help distinguish IM that will progress from IM that is unlikely to progress. Das1 was chosen for this study for its associations not only with incomplete IM but also with complete IM in a cancer setting [22]. Initial validation of the Das1 antibody was accomplished with IHC staining of complete and incomplete IM samples (Fig. 2B).

Serial sections of the IM samples used for the CD10 experiment were stained with Das1 and were scored using the same criteria. Das1 had a low sensitivity of 29.2% but a high specificity of 91.3% for detection of incomplete IM glands in both cohorts combined (Table 1). PPV and NPV of Das1 for incomplete IM glands were 71.8% and 63.0%, respectively. After separation of the cohorts based on potential risk of progression, Das1 continued to demonstrate a low sensitivity across both cohorts (28.6% and 29.3% in IM-GC and IM + GC patients respectively) but a high specificity (98.3% and 85.1% in IM-GC and IM + GC patients respectively).

### Logistic regression model using CD10 and Das1 staining

**Fig. 3:**
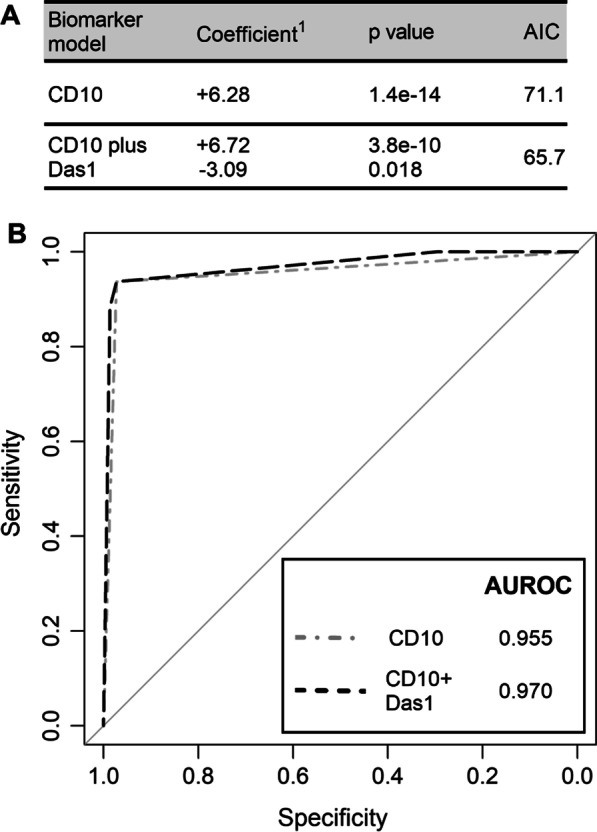
Logistic regression models comparing CD10 with combined CD10 and Das1 staining for complete IM glands. **A** Comparison of CD10 IHC staining on its own and CD10 combined with Das1 IHC staining for complete IM glands using a logistic regression model. ^1^Coefficient shows direction and relative change per unit increase. AIC: Akaike Information Criterion. **B** Receiver Operating Characteristic curves and Areas Under Receiver Operating Characteristic (AUROC) of the logistic regression models. A total of 185 glands with known CD10 and Das1 status from IM-GC and IM + GC patient samples were used together with the glm function in R to create the logistic regression models. The pROC package in R was used to create the graph

To determine whether combined CD10 and Das1 staining could help improve identification of single complete IM glands, logistic regression modelling was performed comparing CD10 on its own with combined CD10 and Das1 staining (glm in R). In the model with CD10 on its own, a highly significant positive association with complete IM glands was observed as expected (Fig. 3A). In the combined model, both CD10 (positive) and Das1 (negative) had a significant association with complete IM glands. The Akaike Information Criterion decreased when Das1 staining status was added to the model suggesting an overall improvement. This was further confirmed by an increase in AUROC observed in the combined model (Fig. 3B). The addition of Das1 offered a small but significant improvement for the detection of complete IM and, by inference, an IM that has lower propensity to progress to cancer.

### Das1 is associated with the incomplete subtype of IM

**Fig. 4:**
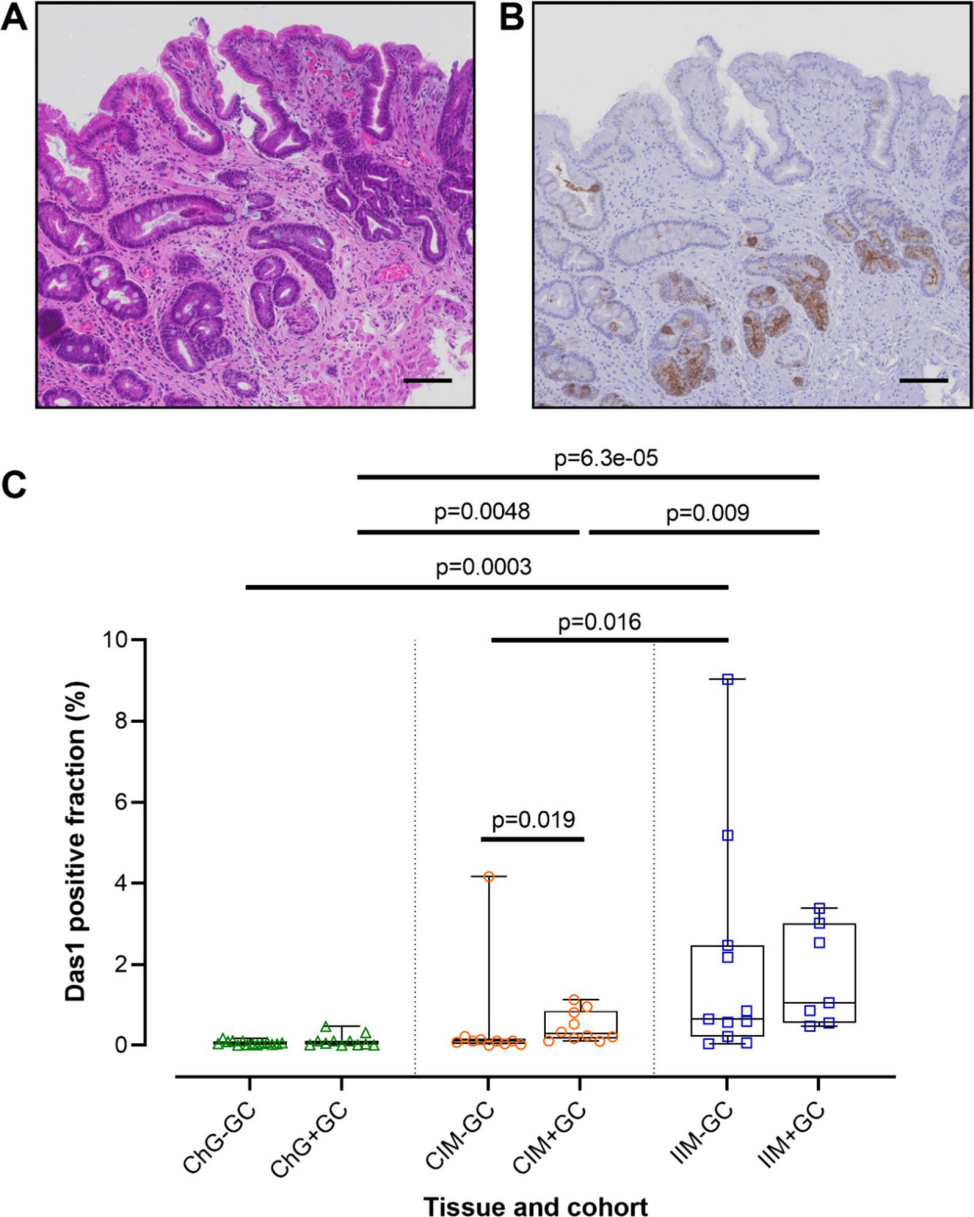
Das1 staining in IM-GC and IM + GC samples. **A** H&E stain of IM tissue, **B** Das1 stains the lower parts of IM glands and **C** digital quantification of Das1 staining for IM-GC (ChG-GC, n = 14; CIM-GC, n = 10; IIM-GC, n = 11) and IM + GC (ChG + GC, n = 11; CIM + GC, n = 10; IIM + GC, n = 7) tissue samples. Statistical analysis carried out using Mann–Whitney test with exception the comparison of CIM + GC with IIM + GC samples where an unpaired t test was used. Scale bars: 100 μm

Das1 staining was often observed in the lower parts of IM glands (Fig. 4A, B). Given that the criteria for single gland analysis was restricted to the quantitation of staining in the top half of the glands, this may explain the low number of IM glands with positive staining. Thus, to determine whether Das1 staining across all parts of IM glands was associated with the incomplete IM subtype, regions rich in IM glands and adjacent ChG as control tissue were digitally quantified for positive staining in patients of both the IM-GC and the IM + GC cohorts (Additional file 1: Figure S3).

Analysis of Das1 staining in the IM-GC cohort showed no staining in ChG, little staining in complete IM but significantly more staining in incomplete IM (*p* = 0.0003 and *p* = 0.016 compared to ChG and complete IM respectively, as determined using the Mann–Whitney test) (Fig. 4C). A single complete IM sample with considerable Das1 staining (4.2% positive staining of IM tissue) was found to be an outlier as determined using the ROUT method. Interestingly this was the only complete IM sample that differed in subtype diagnosis between the original H&E section by the in-house pathologist following endoscopy (incomplete IM) and a second H&E section from the same formalin block cut directly prior to the commencement of the current study (complete IM) (Additional file 1: Table S2, sample N4S2). Gastric IM often consists of interspersed glands with differing subtype thus sections cut from a FFPE block at different levels of depth may differ in IM subtype diagnosis. However, the high levels of positive Das1 staining observed only in this complete IM sample suggest that CEP is likely a marker of local instability normally associated with incomplete IM.

In IM + GC patients, complete IM showed significant more Das1 staining than ChG (*p* = 0.0048, Mann–Whitney test) (Fig. 4C). Again, incomplete IM showed a higher percentage of Das1 staining compared to complete IM (*p* = 0.009, unpaired t test).

### Das1 staining is associated with complete IM in IM + GC samples

Given that adjacent non-malignant tissues from patients with cancer have been shown to be of a more molecularly advanced nature than the same histological tissue type in patients without cancer [35, 36], a comparison was performed of Das1 staining between IM-GC (“early IM lesions”) and IM + GC (“advanced IM lesions”) samples (Fig. 4C). This comparison would allow for the assessment of Das1 staining as a progression risk biomarker. Das1 positive staining in incomplete IM did not differ between IM-GC and IM + GC samples. However, complete IM tissues in IM + GC samples showed a significant increase in Das1 staining compared to those in IM-GC samples (*p* = 0.019, Mann–Whitney test). Overall, these findings suggested that Das1 staining is associated with a more “advanced type” of complete IM (IM + GC cohort) but does not change between “early” and “advanced” incomplete IM.

## Discussion

Several studies have shown a clear association between incomplete IM and a greater risk of progression to GC, but the benefits of reporting IM subtypes in pathology reports is still unclear [15–17]. Currently the BSG guidelines do not recommend reporting as subtyping IM is considered a subjective exercise and thus not consistently reproducible [18]. An ideal biomarker would allow the objective subtyping of IM into complete and incomplete IM subtypes and/or low- and high-risk IM, possibly both.

Previous studies have attempted to identify markers in IM that are associated with greater progression risk, however none have translated into clinical use by pathologists. Schlafen 5 expression has been shown to be correlated with IM patients that progress to GC [37]. Additionally AQP3 was shown to be significantly associated with both IM severity and the incomplete subtype [31].

The current study used gene expression profiling of microarray data to molecularly subtype macro-dissected epithelium enriched IM samples into complete and incomplete IM. Molecular subtyping of IM samples into categories normally associated with histological subtypes has not been previously reported, but differential gene expression and pathway analysis confirmed its potential as complete IM samples were enriched in gene expression and pathways associated with small intestinal brush border, digestion, and metabolism. The overexpression of the gene encoding the chemokine *CCL25*, which is selectively and constitutively expressed in the small intestine epithelium and enables T cell homing via CCR9 binding [38], further suggested that complete IM glands not only mirror biologically the small intestinal crypt/villus but likely recreate a similar T cell microenvironment.

Molecular characterisation of incomplete IM samples did not show pathway enrichment, but multiple overexpressed genes associated with both the colon and GC were detected including *HOXA10* and *HOXA13*. *HOXA10* overexpression in GC patients has been linked with poor survival [39] likely through inhibition of apoptosis [40] and activation of JAK1/STAT3 signalling [41]. *HOXA13* is upregulated in more advanced GC stages and associated with cancer cell invasion suggesting it may play an important role in IM transformation to malignancy [42]. The overall enrichment in GC associated genes including *CLDN1* and *CDH3* [43, 44] suggests that incomplete IM is “primed”, requiring only a small set of genomic changes to initiate the transition to dysplasia. Furthermore the overexpression of the neutrophil chemokine *CXCL5* suggests that incomplete IM is enriched in neutrophil infiltration, previously shown to be 9 and 24 times higher in IM and GC respectively compared to normal tissues [45].

The most significantly enriched brush border gene in the complete IM samples, *MME* and its gene product CD10, was chosen as a candidate biomarker for identifying histologically defined complete IM. With an average AUROC of > 0.94, CD10 was shown to be an outstanding biomarker for complete IM glands [46]. Also, with an average PPV of > 98% and NPV of > 89% across cohorts, lack of CD10 staining could be used to identify incomplete IM glands thus making it a universal biomarker for IM subtyping. This is highly significant as the number *ie* the relative extent of incomplete IM glands is likely to be a more accurate metric of local progression risk to dysplasia, as suggested by Operative Link on Gastric Intestinal Metaplasia (OLGIM) staging with numbers of regions positive for IM [47].

CEP detection by the Das1 antibody in the upper part of the IM gland showed low sensitivity across both cohorts but high to very high specificity for incomplete IM glands, particularly in the IM-GC cohort (above 98%) which is clinically the most relevant (patients that have not progressed to GC). Pathological subtyping of single IM glands using the basal segment was not considered reliable as it is believed to contain the stem cell compartment [48] and does not necessarily express a brush border (personal communication CM). The low sensitivity and low AUROC of Das1 for individual incomplete IM glands suggested that this is unlikely to be useful as a biomarker in a clinical setting on its own. However, the combined use of CD10 and Das1 in a logistic regression model for subtyping complete IM glands did show a small increase in AUROC (0.955 vs 0.970) suggesting added value of using Das1 in a clinical setting for this purpose.

Using digital quantification across total section area, Das1 staining was shown to be more associated with incomplete IM in both the IM-GC and IM + GC cohorts thus confirming previous findings [22, 49]. Finally, Das1 was shown to have potential utility as a progression risk biomarker when used in combination with digital quantification as IM + GC patients with complete IM showed significant more staining than IM-GC patients.

**Fig. 5:**
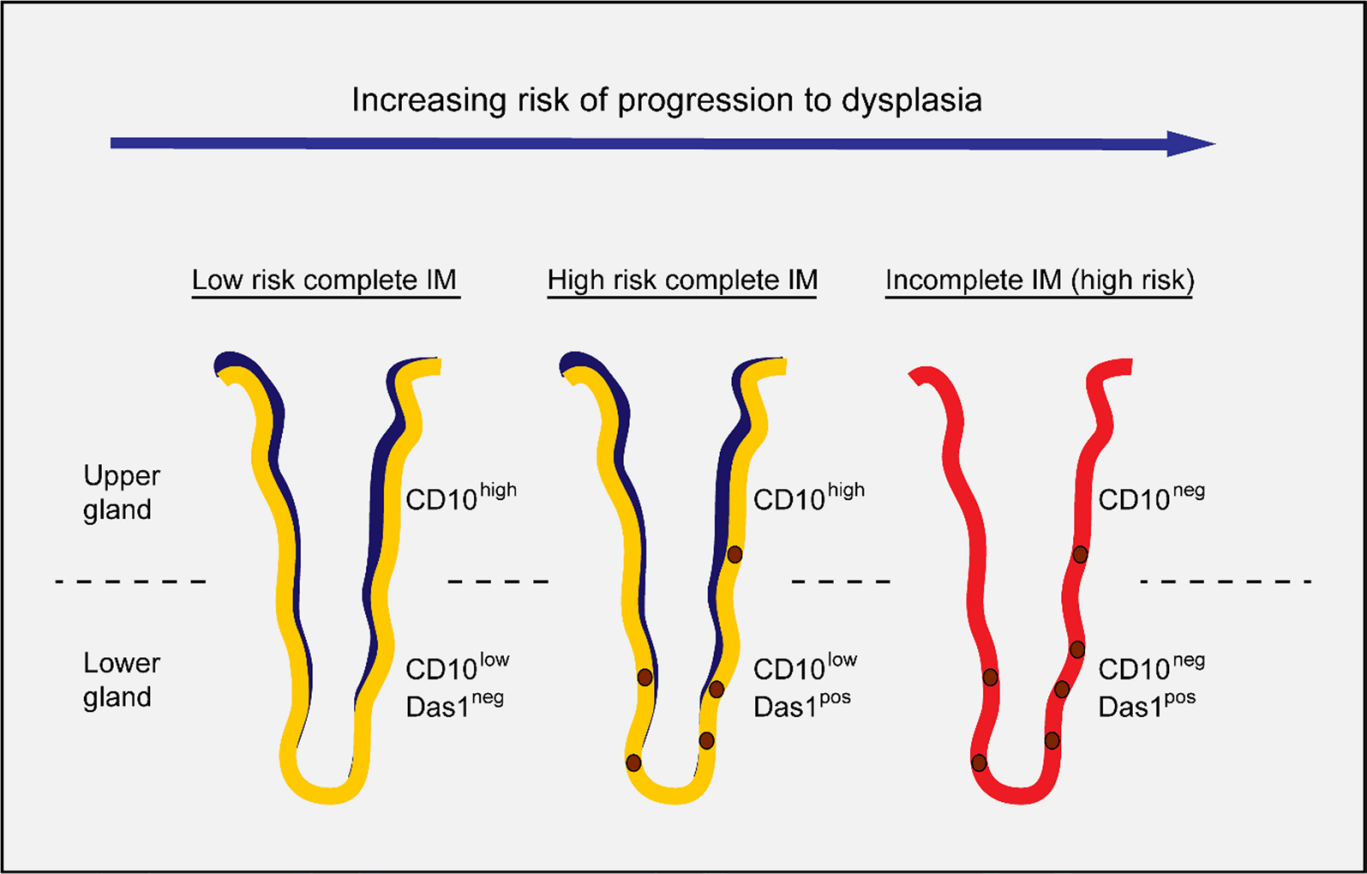
Schematic representation showing combined use of CD10 and Das1 to identify high risk intestinal metaplasia. Schematic model combining CD10 and Das1 staining on IM glands with differing risk of progression. Low risk complete IM is CD10 high in the upper part of the gland and CD10 low in the lower part that includes the stem cell compartment. High risk complete IM is CD10 high in the upper of the gland, CD10 low but also Das1 positive in the lower part of the gland. Incomplete IM is overall CD10 negative but often Das1 positive in the lower part of the gland

Previous studies investigating the relationship of Das1 staining with IM and dysplasia/GC in the same patient have described a consistent positive correlation between IM glands positive for Das1 and distant dysplastic/tumour areas as well as increased staining in both dysplasia and cancer compared to IM [22, 49, 50]. This would suggest that IM glands with high Das1 staining are at the far end of the progression risk spectrum irrespective of subtype. Thus, Das1 may have dual biomarker potential: 1) as a biomarker for the incomplete subtype of IM and 2) as a biomarker delineating increased risk of progression irrespective of IM subtype (Fig. 5).

In combination with a serial anti-CD10 stained section, IM tissue-wide digital quantification of Das1 staining could be used to identify those patients with complete IM which are more likely to progress to the incomplete subtype or directly to dysplasia. First CD10 staining could be used to define areas with complete (positive staining) and incomplete IM (negative staining) glands and their relative abundance. Then Das1 staining could be used to determine whether the tissue contains complete IM glands at greater risk of progression (positive staining).

The strength of the current study lies in its use of IM tissue from two different cohorts of patients with potentially increasing risk of progression to assess the suitability of CD10 and Das1 as biomarkers for complete and incomplete IM. However, it did have several potential limitations including:

- The limited number of tissue samples with appropriate gland orientation. Overall, the “all cohort” analyses provided the most likely accurate results, originating from a total of 18 tissue samples with 215 and 223 individual glands subtyped and scored for CD10 and Das1 staining respectively.
- The use of the top part of IM glands for characterisation had an important effect on the Das1 single gland assessment as a biomarker for incomplete IM. To adjust for this, digital quantification across whole tissue section was carried out which also allowed for an expanded use of patient samples.
- Although promising, the clinical applicability of these two markers warrants further validation in larger cohorts of IM-GC and IM + GC patients. Patients that have been diagnosed with gastric dysplasia (either low or high grade) but that have not yet progressed to gastric cancer could also be included to further strengthen the findings of such a study.

Overall CD10 was shown to be an outstanding biomarker for complete IM and Das1 was shown to have potential as an additional risk-associated biomarker when used in combination with digital imaging quantification. Their clinical use could lead to better patient stratification with improved targeted surveillance of IM patients, ultimately leading to prevention or early detection of GC.

## Supplementary Information

**Additional file 1.** **Table S1**. Clinical details of IM-GC and IM+GC patients used to characterise CD10 and Das1 staining. **Table S2**. Subtyping of IM samples after endoscopy/gastrectomy and again prior to current study. **Figure S1**. Subtyping of individual intestinal metaplasia glands. **Table S3**. Clinical details of IM-GC patients with samples used for gene expression profiling (HG-U133 Plus 2.0) Methods. **Figure S2**. Serial biopsy sections of intestinal metaplasia stained with H&E and CD10 (OPAL™ multiplex immunohistochemistry panel). **Figure S3**. Digital quantification of Das1 staining on intestinal metaplasia glands using an overlay. **Table S4**. Differentially expressed genes (DEGs) in complete and incomplete IM samples. **Figure S4**. Unsupervised hierarchical clustering of all IM-GC samples using differentially expressed gene list. **Table S5**. Significantly enriched KEGG pathways in complete IM determined using ssGSEA with IM-GC samples.

## Data Availability

Data generated or analysed during this study are included in this published article and its supplementary information files. The Affymetrix U133 plus 2 datasets generated and analysed during the current study are available in the Gene Expression Omnibus repository with GEO accession GSE160116 (link: GEO Accession viewer (nih.gov)). Additional raw data files are available from the corresponding author and all R script code used for this study is available as a capsule on codeocean.com (link: https://doi.org/10.24433/CO.8024603.v1).

## Abbreviations

GC: Gastric cancer
IM: Intestinal metaplasia
IM-GC: Patients with intestinal metaplasia but no evidence of gastric cancer
IM + GC: Patients with intestinal metaplasia and concurrent gastric cancer
ChG: Chronic gastritis
BSG: British Society of Gastroenterology
MAPS II: European guidelines for the management of epithelial precancerous conditions and lesions in the stomach
CEP: Colon epithelial protein
MAUGIC: Molecular Analysis for Upper Gastrointestinal Cancer cohort
FFPE: Formalin fixed paraffin embedded tissue
H&E: Haematoxylin and eosin
ssGSEA: Single sample gene set enrichment analysis
KEGG: Kyoto Encyclopedia of Genes and Genomes
PPV: Positive Predictive Value
NPV: Negative Predictive Value
AUROC: Area Under Receiver Operating Characteristic
GEO: Gene Expression Omnibus
