# Supplementary material for: CD10 and Das1: a biomarker study using immunohistochemistry to subtype gastric intestinal metaplasia
Source: BMC Gastroenterol. 2022 Apr 21;22:197. doi: 10.1186/s12876-022-02268-z (PMC9026694; doi:10.1186/s12876-022-02268-z)
Supplement: Supplementary file 1 — Additional file 1. Table S1. Clinical details of IM-GC and IM+GC patients used to characterise CD10 and Das1 staining. Table S2. Subtyping of IM samples after endoscopy/gastrectomy and again prior to current study. Figure S1. Subtyping of individual intestinal metaplasia glands. Table S3. Clinical details of IM-GC patients with samples used for gene expression profiling (HG-U133 Plus 2.0) Methods. Figure S2. Serial biopsy sections of intestinal metaplasia stained with H&E and CD10 (OPAL™ multiplex immunohistochemistry panel). Figure S3. Digital quantification of Das1 staining on intestinal metaplasia glands using an overlay. Table S4. Differentially expressed genes (DEGs) in complete and incomplete IM samples. Figure S4. Unsupervised hierarchical clustering of all IM-GC samples using differentially expressed gene list. Table S5. Significantly enriched KEGG pathways in complete IM determined using ssGSEA with IM-GC samples. [file 12876_2022_2268_MOESM1_ESM.docx]

# ADDITIONAL FILE 1

**Table S1.** Clinical details of IM-GC and IM+GC patients used to characterise CD10 and Das1 staining

| Parameters | IM-GC (n=18) | IM+GC (n=19)^a^ |
| --- | --- | --- |
| Gender |  |  |
| Male | 12 | 13 |
| Female | 6 | 6 |
| Age (median) |  |  |
| Male | 69 (39-86)^b^ | 74 (60-95)^b^ |
| Female | 71.5 (44-79)^b^ | 67 (49-83)^b^ |
| Premalignant tissue |  |  |
| Chronic gastritis | 12 | 27 |
| Intestinal metaplasia | 22 | 33 |
| Matched tumour pathology |  |  |
| Intestinal | - | 14 |
| Diffuse | - | 5 |
| Matched tumour location |  |  |
| Antrum | - | 12 |
| Body | - | 6 |
| GOJ | - | 1 |
| T stage |  |  |
| Tis | - | 4 |
| T1 | - | 3 |
| T2 | - | 3 |
| T3 | - | 8 |
| Unspecified | - | 1 |
| Total n^0^ of IM glands characterised^c^ |  |  |
| CD10 | 96 | 119 |
| Das1 | 81 | 142 |

IM-GC: patients with no evidence of gastric cancer; IM+GC: patients with concurrent gastric cancer; ^a^Cohort included two patients with intra-mucosal gastric cancer from whom tissue was taken from multiple stomach sites and characterised across multiple time points; ^b^Age range; ^c^Glands were selected with the criterion that they contained the upper part of the gland near the surface (Additional file 1: Figure S1).

**Table S2.** Subtyping of IM samples after endoscopy/gastrectomy and again prior to current study

| SampleID | 1^st^ IM subtyping  (post-endoscopy/gastrectomy) | 2^nd^ IM subtyping  (directly prior to study) |
| --- | --- | --- |
| IM-GC |  |  |
| N1S1^a^ | Complete | Complete |
| N1S2^a^ | Complete | Complete |
| N1S3^a^ | Complete | Complete |
| N2S1^b^ | Complete | Complete |
| N2S2^b^ | Complete | Incomplete |
| N3S1 | Complete | Complete |
| N4S1^c^ | Complete | Mixed |
| N4S2^c^ | Incomplete | Complete |
| N5S1 | Mixed | Incomplete |
| N6S1 | Complete | Complete |
| N7S1 | Incomplete | Incomplete |
| N8S1 | Complete | Complete |
| N9S1 | Complete | Mixed |
| N10S1 | Mixed | Mixed |
| N11S1 | Mixed | Mixed |
| N12S1 | Mixed | Incomplete |
| N13S1 | Incomplete | Incomplete |
| N14S1 | Mixed | Incomplete |
| N15S1 | Complete | Complete |
| N16S1 | Complete | Mixed |
| N17S1 | Incomplete | Incomplete |
| N18S1 | Complete | Complete |
| IM+GC | |  |
| P1 | Incomplete | Mixed |
| P2 | Complete | Complete |
| P3 | Incomplete | Incomplete |
| P4 | Complete | Complete |
| P5 | Complete | Complete |
| P6 | Incomplete | Mixed |
| P7 | Mixed | Complete |
| P8 | Complete | Incomplete |
| P9 | Mixed | Mixed |
| P10 | Complete | Complete |
| P11 | Complete | Complete |
| P12 | Complete | Incomplete |
| P13 | Mixed | Complete |
| P14 | Incomplete | Complete |
| P15 | Mixed | Mixed |
| P16 | Incomplete | Complete |
| P17 | Complete | Complete |

Second IM subtyping carried out on same FFPE block as before but from freshly cut sections. ^a^Patient N1: biopsies from 3 different endoscopies and sites (A1, A4, A5) as recommended by the updated Sydney protocol; ^b^Patient N2: biopsies from a single endoscopy (A1, A4); ^c^Patient N4: biopsies from 2 endoscopies (both A1). Mixed and incomplete IM considered of equal risk and pooled together for analysis purposes.

**
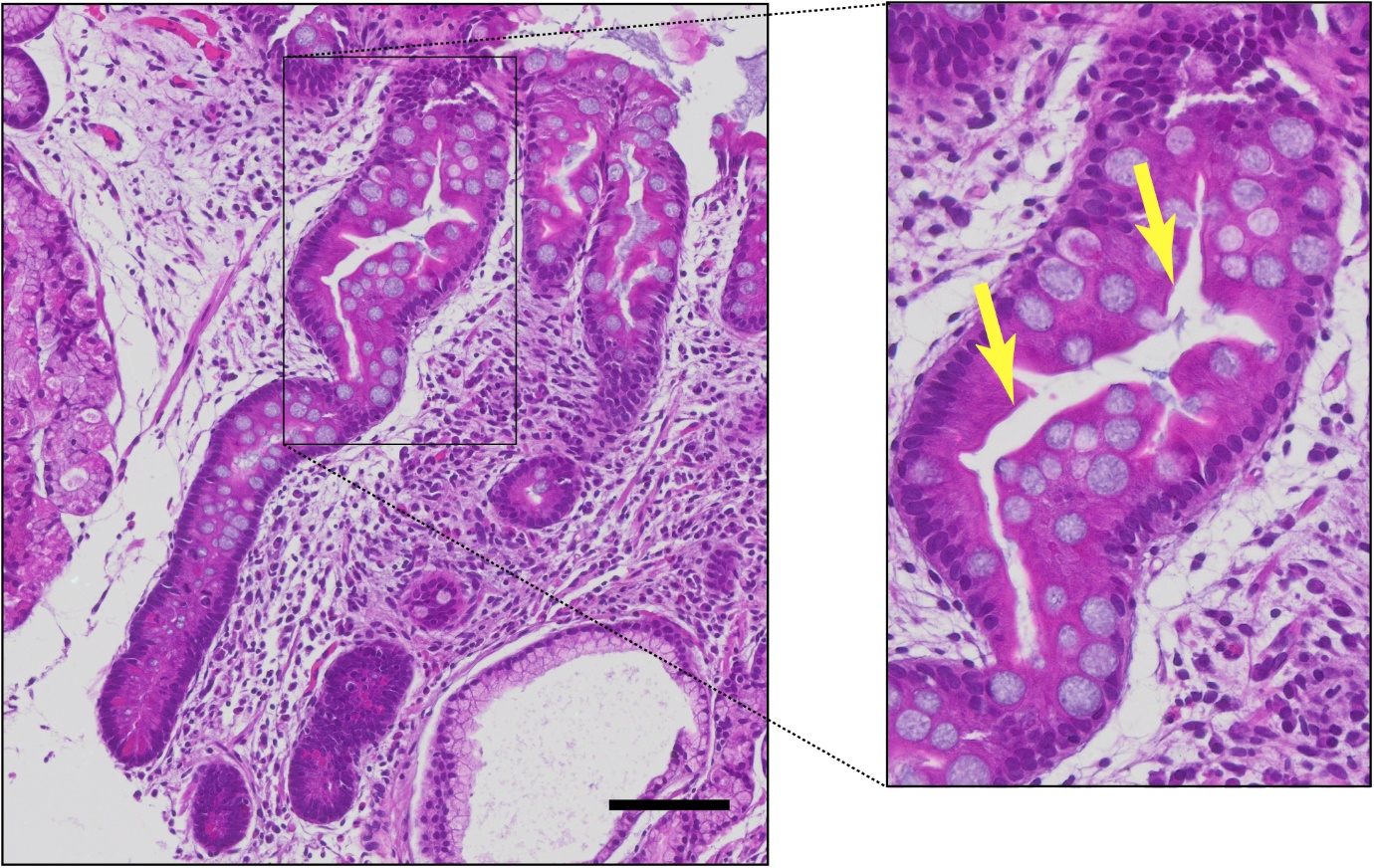
**

**Figure S1.** Subtyping of individual intestinal metaplasia glands.

Image showing individual IM glands, with a single complete IM gland shown in inset, as defined by the presence of a brush border (yellow arrows). Individual IM glands were subtyped using the upper part of the gland and gland morphology and classified as complete (with brush border) or incomplete (absence of brush border and irregular architecture). Scale bar: 100μm.

**Table S3.** Clinical details of IM-GC patients with samples used for gene expression profiling (HG-U133 Plus 2.0)

| CEL file | SampleID | Gender | Age |
| --- | --- | --- | --- |
| 08-0017ANTA.CEL^a^ | S1 | Male | 68 |
| 08-0021BODY2.CEL | S2 | Male | 44 |
| 08-020-02ANTB.CEL | S3 | Male | 32 |
| 09-003-01B4.CEL | S4 | Male | 67 |
| 09-020-02B1.CEL | S5 | Male | 75 |
| 09-024-02B5.CEL^a,b^ | S6 | Male | 69 |
| 09-025-01B1.CEL^b^ | S7 | Male | 84 |
| 09-032-01B3.CEL^b^ | S8 | Female | 79 |
| 09-033-03B3.CEL^b^ | S9 | Male | 59 |
| 10-038-01B1.CEL^b^ | S10 | Male | 39 |
| 10-043-01 B3.CEL | S11 | Female | 82 |
| 2006-0011ANTB.CEL | S12 | Male | 56 |
| 2006-008ANTB.CEL | S13 | Male | 70 |
| 2007-0015ANT.CEL | S14 | Male | 49 |

**^a^**RNA was extracted and profiled from biopsy tissue collected from the same patient at two different hospital visits. ^b^Biopsy tissue acquired from these patients was also used in the IHC staining part of the study. The complete Affymetrix microarray dataset can be accessed using GEO accession number: GSE160116 (https://www.ncbi.nlm.nih.gov/geo).

**Methods.** Immunohistochemical staining with anti-CD10 antibody

Single stain immunohistochemistry with anti-CD10 (clone 56C6, Abcam, catalogue number: ab951) consisted of overnight incubation with the primary antibody (dilution 1:50) at 4^0^C followed by incubation with the EnVision+ System/anti-mouse HRP reagent and visualisation with DAB chromogen.

The single gland CD10 study was carried out using immunofluorescence (IF) staining with the OPAL™ reagent 650 for visualisation purposes as part of an optimised multiplex IHC panel to detect T cell subsets (part of another on-going study and mostly based on the panel described in (26)) using the OPAL™ 7-Color IHC kit (PerkinElmer, cat# NEL811001KT). Briefly 4μm FFPE sections were cyclically incubated followed by an antigen retrieval step with anti-CD4 (clone 4B12, ThermoFisher Scientific, cat# MA5-12259), anti-CD8 (clone 4B12, ThermoFisher Scientific, cat# MA1-80231), anti-FOXP3 (Bio SB, cat# BSB 6761), anti-CD3 (clone SP7, Spring Bioscience, catalogue number: M3074), anti-CD10, anti-AE1AE3 (AE1/AE3, Leica Biosystems, cat# NCL-L-AE1/AE3) and Dapi (PerkinElmer) for 30 minutes at room temperature (RT), washed and incubated with the species appropriate HRP-labelled secondary antibody (10 minutes at RT) and visualised with OPAL™ reagents diluted in 1x amplification diluent (10 minutes at RT). Slide scanning was performed on a VECTRA® automated quantitative pathology imaging system and visualisation was obtained through multi-spectral unmixing with the use of inForm® software (both PerkinElmer).

**
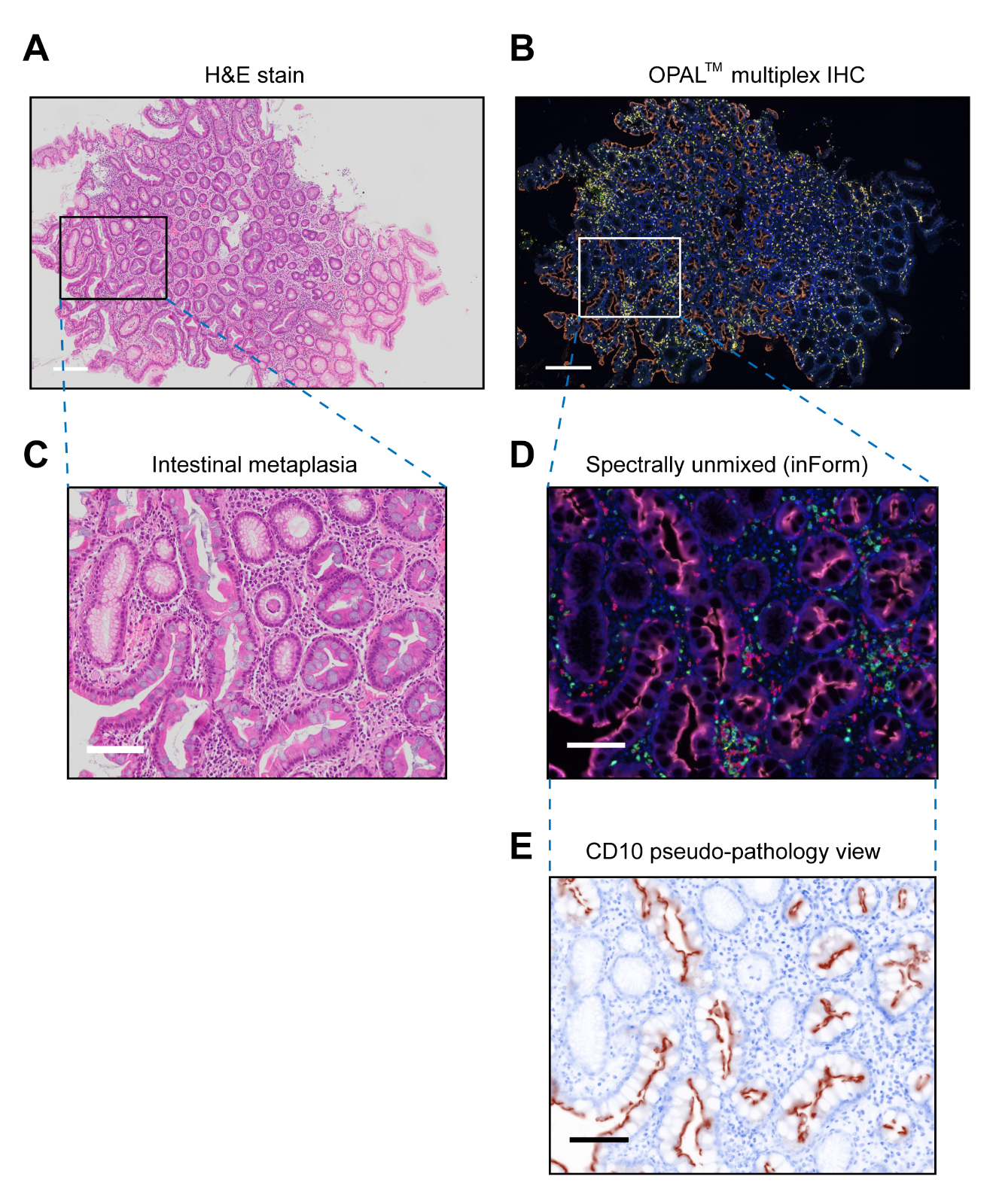
**

**Figure S2.** Serial biopsy sections of intestinal metaplasia stained with H&E and CD10 (OPAL™ multiplex immunohistochemistry panel).

Serial FFPE sections at 4μm were used for A: H&E staining and B: OPAL™ multiplex immunohistochemistry (IHC) staining; C: inset of H&E stained section showing IM, D: matched spectrally unmixed image using inForm® software (PerkinElmer) from the multiplexed IHC scanned slide and E: pseudo-pathology view for CD10 used for single gland scoring. Scale bars: 200µm for A and B, 100µm for C, D and E.

**
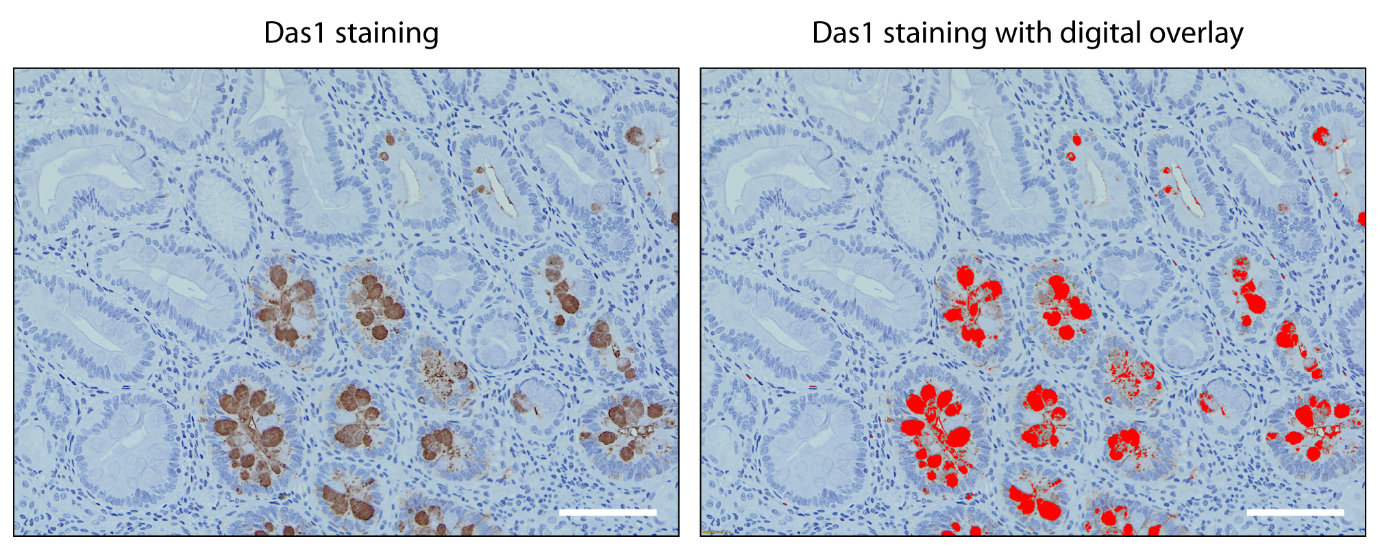
**

**Figure S3.** Digital quantification of Das1 staining on intestinal metaplasia glands using an overlay.

Digital quantification of Das1 staining was carried out on high resolution .vsi files using the cellSens Dimension software (Olympus). Fraction area of each region of interest (ROI) positive for Das1 staining was measured by using first the “threshold tool” with R-G-B threshold detection parameters kept constant for all images and second with the “Count and Measure on ROI” tool. All result files were exported in csv format for further analysis. Scale bar: 100μm.

**Table S4.** Differentially expressed genes (DEGs) in complete and incomplete IM samples

| **Symbol** | **Name** | **logFC** | **AveExpr** | **adj.P.Val** |
| --- | --- | --- | --- | --- |
| High in CIM^a^ | |  |  |  |
| RBP2 | Retinol Binding Protein 2 | -5.36 | 10.73 | 1.7e-02 |
| MME | Membrane Metalloendopeptidase | -4.08 | 7.22 | 3.9e-03 |
| APOA4 | Apolipoprotein A4 | -3.96 | 9.34 | 4.6e-02 |
| CCL25 | C-C Motif Chemokine Ligand 25 | -3.88 | 7.29 | 9.2e-03 |
| SLC2A5 | Solute Carrier Family 2 Member 5 | -3.74 | 7.60 | 2.4e-02 |
| SLC2A2 | Solute Carrier Family 2 Member 2 | -3.17 | 6.40 | 8.1e-03 |
| XPNPEP2 | X-Prolyl Aminopeptidase 2 | -3.05 | 7.29 | 1.7e-02 |
| MGAM | Maltase-Glucoamylase | -3.00 | 7.69 | 2.4e-02 |
| SEMA6D | Semaphorin 6D | -2.36 | 5.89 | 1.9e-02 |
| SLC5A1 | Solute Carrier Family 5 Member 1 | -2.16 | 8.87 | 3.7e-02 |
| KHK | Ketohexokinase | -1.98 | 8.93 | 4.6e-02 |
| PCK2 | Phosphoenolpyruvate Carboxykinase 2 | -1.73 | 10.09 | 4.7e-02 |
| KLK11 | Kallikrein Related Peptidase 11 | -1.67 | 7.65 | 3.4e-02 |
| TTTY14 | Testis-Specific Transcript, Y-Linked 14 | -1.49 | 5.55 | 4.6e-02 |
| SFRP5 | Secreted Frizzled Related Protein 5 | -1.34 | 6.10 | 1.7e-02 |
| SLC37A4 | Solute Carrier Family 37 Member 4 | -1.24 | 8.56 | 4.6e-02 |
| ADRB2 | Adrenoceptor Beta 2 | -0.90 | 6.04 | 3.2e-02 |
| NUP210 | Nucleoporin 210 | -0.85 | 7.12 | 1.7e-02 |
| High in IIM^b^ | |  |  |  |
| HOXA10 | Homeobox A10 | 3.09 | 6.60 | 2.5e-03 |
| HOXA13 | Homeobox A13 | 2.80 | 5.74 | 1.5e-02 |
| CXCL5 | C-X-C Motif Chemokine Ligand 5 | 2.49 | 5.48 | 1.7e-02 |
| CLDN1 | Claudin 1 | 2.09 | 7.28 | 1.9e-02 |
| F5 | Coagulation Factor V | 1.89 | 7.25 | 3.2e-02 |
| CDH3 | Cadherin 3 | 1.69 | 7.62 | 5.6e-03 |
| CTSV | Cathepsin V | 1.61 | 7.44 | 1.9e-02 |
| MUC4 | Mucin 4 | 1.51 | 4.78 | 3.5e-02 |
| CPD | Carboxypeptidase D | 1.26 | 8.01 | 4.5e-02 |
| SEMG1 | Semenogelin 1 | 1.16 | 4.19 | 2.7e-02 |
| RSPH1 | Radial Spoke Head Component 1 | 1.11 | 7.17 | 2.0e-02 |
| FAM3D | FAM3 metabolism regulating signaling molecule D | 0.69 | 10.16 | 3.0e-02 |

^a^complete IM; ^b^incomplete IM. Differentially expressed genes were defined as log2 fold change >0.6 or <-0.6 with adjusted p<0.05 using the Benjamini-Hochberg method for multiple test correction.





**Figure S4.** Unsupervised hierarchical clustering of all IM-GC samples using differentially expressed gene list.

Gene expression heatmap showing unsupervised clustering of all IM-GC samples based on previously obtained differentially expressed gene list. CIM: complete IM, IIM: incomplete IM and MIM: mixed IM. Genes with average expression level <5 removed (SEMG1 and MUC4). Pheatmap package in R used. Expression levels have been standardised (centered and scaled) within rows for visualization. Legend shows z score.

**Table S5.** Significantly enriched KEGG pathways in complete IM determined using ssGSEA with IM-GC samples^a^

| Pathway | Adj.P.Val |
| --- | --- |
| Galactose Metabolism | 0.031 |
| Glycine Serine And Threonine Metabolism | 0.031 |
| Glycerolipid Metabolism | 0.031 |
| Arachidonic Acid Metabolism | 0.031 |
| Linoleic Acid Metabolism | 0.031 |
| Retinol Metabolism | 0.031 |
| Metabolism Of Xenobiotics By Cytochrome P450 | 0.031 |
| Drug Metabolism Cytochrome P450 | 0.031 |
| Drug Metabolism Other Enzymes | 0.031 |
| Abc Transporters | 0.031 |
| Renin Angiotensin System | 0.031 |
| Hematopoietic Cell Lineage | 0.031 |
| Maturity Onset Diabetes Of The Young | 0.031 |
| Starch And Sucrose Metabolism | 0.047 |
| Glycerophospholipid Metabolism | 0.047 |
| Alpha Linolenic Acid Metabolism | 0.047 |
| Leishmania Infection | 0.047 |

^a^ssGSEA carried out on complete IM (n=6) and incomplete IM (n=6) samples using the GSVA package in R. Statistical significance was calculated using the Wilcoxon rank sum test with Benjamini-Hochberg correction. KEGG pathways database (version 7.1) downloaded from the Molecular Signatures Database (www.gsea-msigdb.org/gsea/msigdb/index.jsp).
